# Increased cFLIP expression in thymic epithelial tumors blocks autophagy via NF-κB signalling

**DOI:** 10.18632/oncotarget.15929

**Published:** 2017-02-06

**Authors:** Djeda Belharazem, Albert Grass, Cornelia Paul, Mario Vitacolonna, Berthold Schalke, Ralf J. Rieker, Daniel Körner, Philipp Jungebluth, Katja Simon-Keller, Peter Hohenberger, Eric M. Roessner, Karsten Wiebe, Thomas Gräter, Thomas Kyriss, German Ott, Peter Geserick, Martin Leverkus, Philipp Ströbel, Alexander Marx

**Affiliations:** ^1^ Institute of Pathology and Medical Research Center (ZMF), University Medical Centre Mannheim, University of Heidelberg, Mannheim, Germany; ^2^ Department of Thoracic Surgery, University Medical Centre Mannheim, University of Heidelberg, Mannheim, Germany; ^3^ Department of Neurology, University of Regensburg, Regensburg, Germany; ^4^ Institute of Pathology, University Hospital Heidelberg, University of Heidelberg, Heidelberg, Germany; ^5^ Institute of Pathology, University Hospital Erlangen, Friedrich-Alexander University Erlangen-Nuremberg, Erlangen, Germany; ^6^ Department of Thoracic Surgery, Thorax Clinic, University of Heidelberg, Heidelberg, Germany; ^7^ Department of Thoracic Surgery, University of Münster, Münster, Germany; ^8^ Department of Thoracic Surgery, Clinic Löwenstein, Löwenstein, Germany; ^9^ Department of Thoracic Surgery, Clinic Schillerhöhe, Robert-Bosch-Hospital, Gerlingen, Germany; ^10^ Department of Clinical Pathology, Robert-Bosch-Hospital, Stuttgart, Germany; ^11^ Dr. Margarete Fischer-Bosch Institute for Clinical Pharmacology, Stuttgart, Germany; ^12^ Department of Dermatology, Venereology, and Allergology, Medical Faculty Mannheim, University of Heidelberg, Mannheim, Germany; ^13^ Department for Dermatology and Allergology, University Hospital Aachen, RWTH Aachen, Aachen, Germany; ^14^ Institute of Pathology, University Medical Center Göttingen, University of Göttingen, Göttingen, Germany

**Keywords:** thymic tumors, cFLIP, NF-κB, senescence, autophagy

## Abstract

The anti-apoptotic cellular FLICE-like inhibitory protein cFLIP plays a pivotal role in normal tissues homoeostasis and the development of many tumors, but its role in normal thymus (NT), thymomas and thymic carcinomas (TC) is largely unknown.

Expression, regulation and function of cFLIP were analyzed in biopsies of NT, thymomas, thymic squamous cell carcinomas (TSCC), thymic epithelial cells (TECs) derived thereof and in the TC line 1889c by qRT-PCR, western blot, shRNA techniques, and functional assays addressing survival, senescence and autophagy. More than 90% of thymomas and TSCCs showed increased cFLIP expression compared to NT. cFLIP expression declined with age in NTs but not in thymomas. During short term culture cFLIP expression levels declined significantly slower in neoplastic than non-neoplastic primary TECs. Down-regulation of cFLIP by shRNA or NF-κB inhibition accelerated senescence and induced autophagy and cell death in neoplastic TECs.

The results suggest a role of cFLIP in the involution of normal thymus and the development of thymomas and TSCC. Since increased expression of cFLIP is a known tumor escape mechanism, it may serve as tissue-based biomarker in future clinical trials, including immune checkpoint inhibitor trials in the commonly PD-L1^high^ thymomas and TCs.

## INTRODUCTION

Thymomas and thymic carcinomas (TC) are rare thymic epithelial tumors with poorly understood pathogenesis and no curative options beyond surgery [1]. Thymomas are separated into WHO type A, AB, B1, B2 and B3 histological subtypes [2]. Thymomas variably maintain thymic functions, including functionally compromised thymopoiesis that predisposes to autoimmunity and immunodeficiencies [3], and are used to study thymic function and immunosenescence in humans [4]. Thymic squamous cell carcinoma (TSCC) is the most common TC subtype, behaves more malignant than thymomas [5] and lacks thymus-specific functions. Genetic alterations of TSCC and thymomas are significantly different from those of squamous cell carcinomas of head, neck and lung [6]. This reflects the enigmatic etiology of thymomas and TCs compared to the known environmental triggers of most upper aero-digestive tract cancers. A clue to the oncogenesis of thymomas and TSCCs could be their increasing incidence with age that parallels thymic involution. This and the known potential of senescence to eliminate pre-cancerous cells make us hypothesize, that genetic and epigenetic alterations that interfere with thymic involution could contribute to the development of thymomas and TSCCs. Since thymic epithelial cells (TECs) show turnover in the normal thymus (NT) up to old age [7], thymic involution can formally be explained by TEC death outweighing proliferation, but underlying molecular mechanisms are poorly understood [8]. Along this line, we previously found that an inhibitor of the *intrinsic/mitochondrial* apoptotic pathway, BIRC3 shows increased expression in TSCC but not in thymomas compared to NT [9]. By contrast, we report here that there is increased expression of cellular FLICE-like inhibitory protein (cFLIP), a key inhibitor of the *extrinsic*, TNF*α*-, FAS-L- and TRAIL-driven apoptosis pathway [10], in both thymomas and TSCCs compared to NT. cFLIP is expressed in various splice variants (mainly cFLIP_L_ (cFLIPlong) and cFLIP_S_ (cFLIPshort)) in normal tissues and many tumors [11] and is regulated by various factors, including NF-κB [11]. Apart from blocking apoptosis, cFLIP_L_ plays a role in autophagy suppression [12], which can interfere with cell death as well. Therefore, we compared cFLIP and NF-κB expression, and the functional relevance of cFLIP over-expression and NF-κB blockade on TEC survival and autophagy in resection specimens and primary cell cultures of NT, thymomas, TCs and the TC cell line 1889c [13].

## RESULTS

### Thymomas and TSCCs show increased cFLIP expression compared to NT *in vivo*

**Figure 1:**
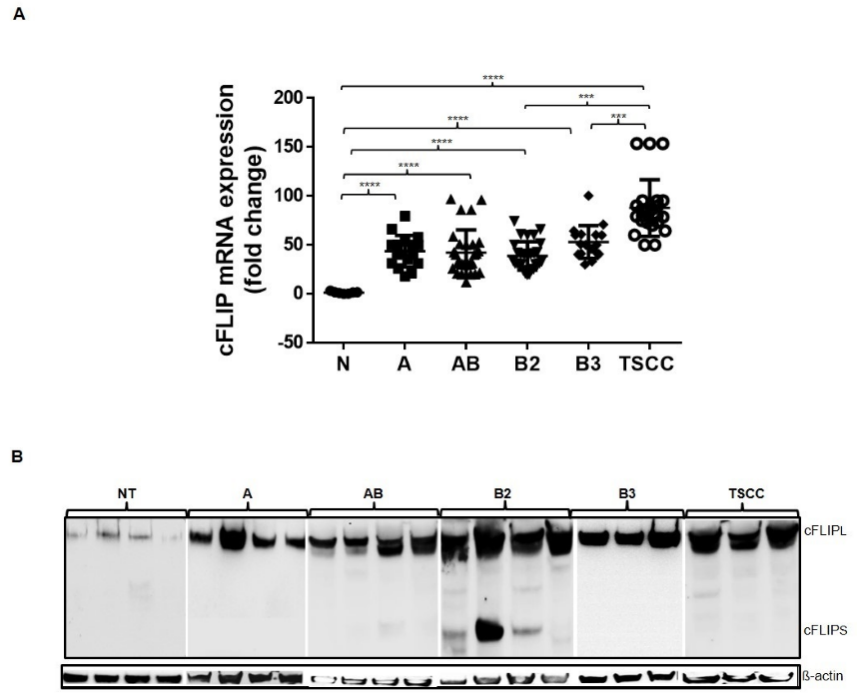
cFLIP mRNA and protein expression analysis **A.** mRNA expression by Q-PCR in 67 thymoma and 15 TSCC compared to 5 normal thymuses (NT). The applied primers recognize sequences shared by cFLIP_L_ and cFLIP_S_^****^p<10^-4^, ^***^p=0.0002 and p=0.0009). **B.** Expression of cFLIP_L_ and cFLIP_S_ protein in 4 NT and 15 thymomas (4 type A, AB, B2 and 3 type B3) and 3 TSCC. ß-actin served as loading control.

We found significantly (p<0.001) higher expression levels of cFLIP RNA and protein in whole tissue extracts of thymomas (n=67) and TSCCs (n=15) than in NT (n=15) (Figure 1A). RNA levels varied within each histological tumor subtype (range: 3-180 fold compared to the average level of 15 NTs). Between the thymoma subtypes, RNA und protein levels were not significantly different. However, RNA levels were significantly higher in TSCCs compared to type A, AB and B2 thymomas (p <0.0001) and B3 thymomas (p=0.0009). There was no significant correlation between tumor stage and cFLIP mRNA levels. cFLIP_L_ represented the only expressed isoform in thymoma subtypes excepted in B2 thymomas that showed both cFLIP_S_ and cFLIP_L_ expression (Figure 1B). When cFLIP RNA levels were normalized for cytokeratin 19 (CK19) expression levels (as surrogate marker of epithelial cell content), the difference between thymomas and TSCCs disappeared, while the individual cFLIP/CK19 ratios of the different thymic tumor subtypes (mean ratios 1,58 - 2,0) were still significantly higher than the ratio of NTs (ratio 0,33 +/-0,13) (Supplementary Figure 2 and Table 1). Higher RNA expression was accompanied by higher protein expression in thymomas and TSCCs compared to NT on western blot (Figure 1B). cFLIP_L_ splice variant is more expressed in all thymomas except in three B2 subtype tumors (Figure 1B). Expression levels of cFLIP were not different in females and males within each cohort of NTs and thymic tumors (not shown).

**Table 1:** Characteristics of 82 tumor patients and tissues studied for cFLIP compared to 20 adult cardiac surgery patients with normal thymus (NT)

Diagnosis	N	Age range (y)	Sex (m:f)	Stage (I-IV)	MG+ (%)	Follow-up
Thymoma						
Type A	8	36-87	05:03	I (n=3)II (n=5)	1 (12.5%)	n.k
Type AB	22	26-79	08:14	I (n=9)II (n=12)	9 (40.9%)	n.k
Type B2	18	21-81	08:10	I (n=9)	8 (44.4%)	Partially known
				II (n=4)		2: dead
				III (n=2)		2: alive
				IV (n=3)		
Type B3	19	41-76	09:10	I (n=6)	4 (21.05%)	Partially known
				II (n=4)		1: dead
				III (n=4)		11: alive
				IV (n=5)		
TSCC	15	32-74	10:05	I (n=1)	0	Partially known
				II (n=5)		7: dead
				III (n=4)		
				IV (n=5)		
NT	20	28-82		—	—	—

Thymoma type A, AB, B2, and B3 (WHO classification); TSCC, thymic squamous cell carcinoma; MG+ (%), percentage of patients with Myasthenia gravis; n.k., not known; stage, according to Masaoka-Koga (2).

### cFLIP expression *in vivo* declines with age in NT but not in thymomas and TSCCs

In NTs (n=15) cFLIP RNA expression levels declined with age from 5,27+/-0,9 (age 28-35 years, n=5) through 1,33+/-0,18 (40-57 years, n=6, p=0,0013) to 0,166+/-0,10 (61-82 years, n=3, p=0,0062) (Supplementary Figure 3A). By contrast, no age-related decline of cFLIP expression levels was observed in thymomas and TSCCs (Supplementary Figure 3B)

### cFLIP expression declines more slowly in neoplastic than normal pTECs on prolonged cell culture

**Figure 2:**
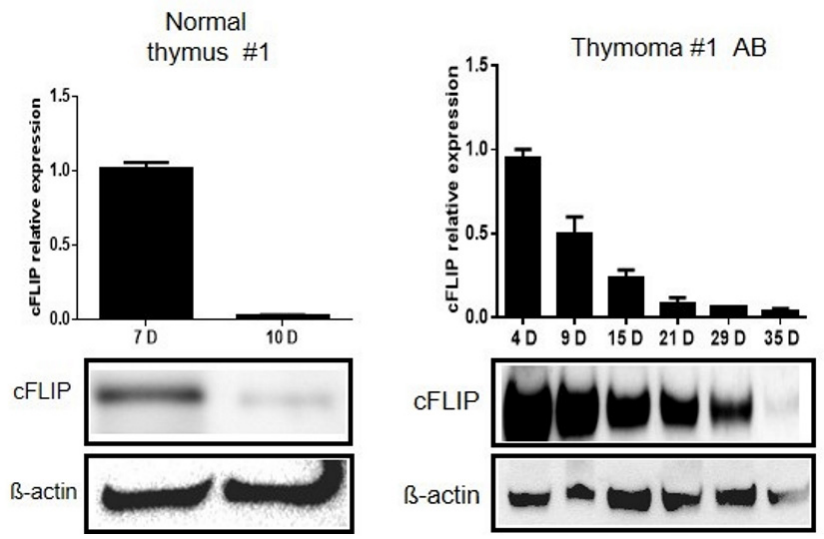
Slower decline of cFLIP mRNA and protein levels in thymoma primary epithelial cells compared to primary epithelial cells from normal thymus Higher cFLIP expression levels in primary thymic epithelial cells (pTECs) from a type AB thymoma compared to a normal thymus (NT). Subconfluent pTECs were trypsinized for passaging at the indicated culture times after surgery (4-35 days) and RNA and protein levels were analyzed using real time PCR and western blot analysis, respectively. D: days of cell or tissue culture after surgery. The mRNA quantification result represents the mean +/- SEM of three independent experiments. Comparable results were obtained with pTECs from 3 other NTs and 4 other thymomas (Supplementary Figure 3).

EpCam(+) primary thymic epithelial cells (pTECs) established from resection specimens of thymomas showed higher cFLIP mRNA and protein levels than pTECs established from NTs (Figure 2 and Supplementary Figure 4) at the time of sub-confluence and first passaging. Subsequently, cFLIP expression decreased more rapidly in pTECs from NTs (n=4) than in 3 of 4 investigated neoplastic pTECs (Figure 2). This was accompanied by the failure to split pTECs derived from NTs more than once under our cell culture conditions. The time-dependent decline of cFLIP levels in neoplastic and non-neopl+astic pTECs is not a general feature of ex vivo established cell cultures: a prostate cancer primary cell culture and several primary fibroblast cultures derived from different tumors did not show a drop in cFLIP expression on prolonged cell culture (data not shown).

### Delayed decline of cFLIP expression in neoplastic pTECs is associated with delayed start of senescence

**Figure 3:**
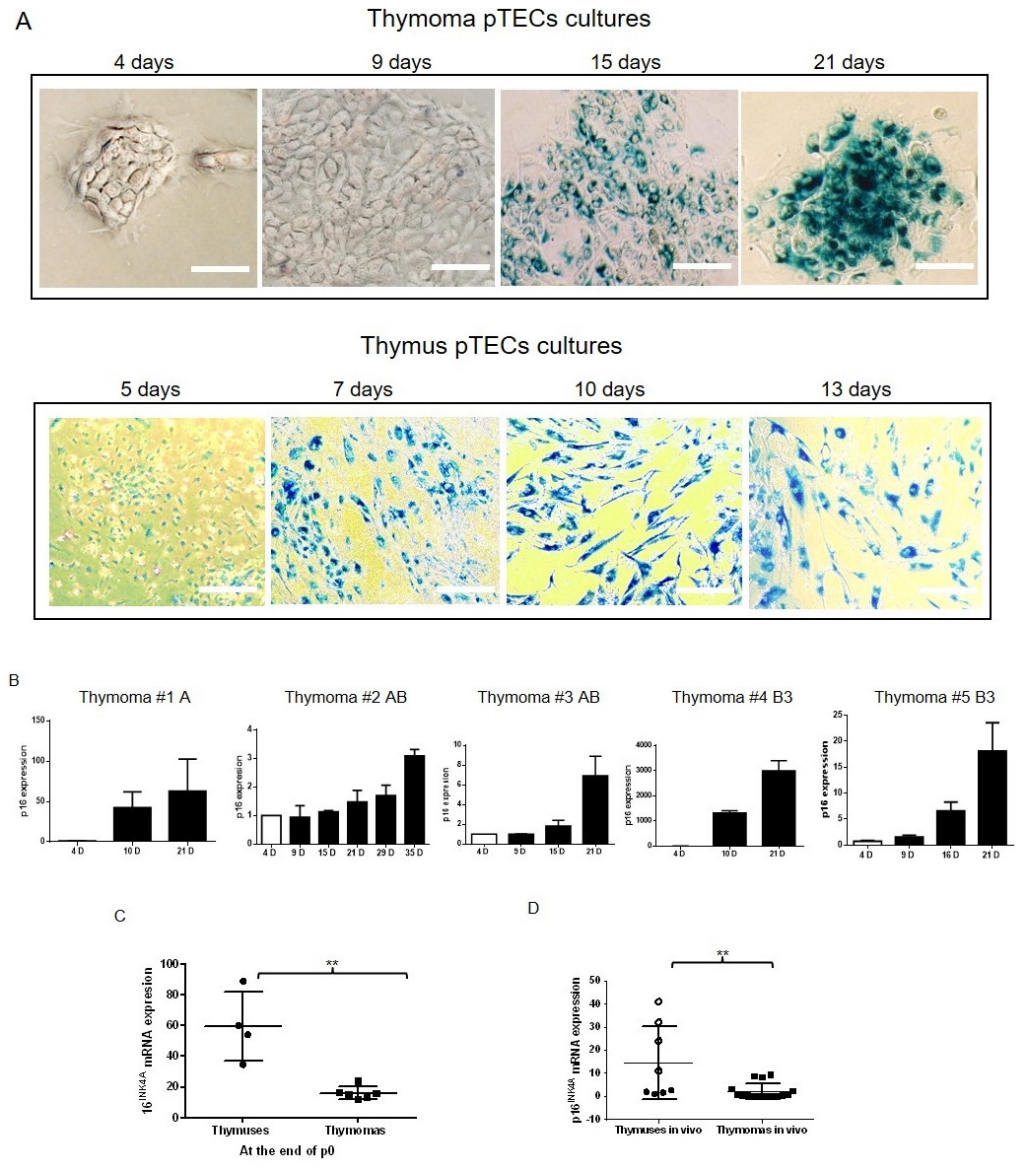
Senescence detection in thymoma and NT pTECs by X-Gal staining **A.** Senescence of primary thymic epithelial cells (pTECs) as revealed by X-Gal staining starts consistently earlier in normal thymic (NT) pTECs than pTECs from thymomas. The number of passages is indicated in brackets (P0, primary culture). **B.** Progressive increase of p16^INK4A^ expression in thymomas was detected by real time PCR at the time of pTEC passaging; expression levels were normalized to the expression at the end of 4 days (4D) culture that was set as 1. **C.** Comparison of p16^INK4A^ expression in pTECs from normal thymuses (NT; n=4; primary passage, p0) and thymomas (n=6; 1 type A, 1 type AB, 1 type B2 and 3 type B3; passages p0-p5). **D.** For comparison, *in vivo* p16^INK4A^ expression levels in whole tissue extracts of NTs (n=8; age 28-47 years) and A, AB and B3 thymomas (n=16; age 26-79 years) are shown. The results represent the mean +/- SEM. The results in figure B represent experiments in triplicates. “D”: days. The dark circles in figure C represent NTs of 28 and 29 year-old patients, light circles represent NTs of 46 and 47 year-old patients.

While cFLIP expression decreased in pTECs during cell culture (see above), X-Gal staining intensities increased over time (Figure 3A). This suggests progressive senescence *in vitro*. In pTECs from thymomas, senescence became recognizable after 15 days in culture, and RNA expression of p16^INK4A^ (a senescence-associated gene) started to rise progressively on day 10 to 15 after surgery and lasted till the 3^rd^ to 5^th^ passage (Figure 3B). By contrast, pTECs from NTs showed X-Gal staining positivity and increased p16^INK4A^ RNA levels already after 5-7 days in primary culture (Figure 3C). Furthermore, p16^INK4A^ RNA levels of whole tissue extracts of NTs (age 12-29 years) were significantly higher than levels in thymomas (p= 0.0014) (Figure 3D). Taken together, the negative correlation between cFLIP expression levels and levels of senescence markers in pTECs *in vitro*, and the increased cFLIP levels and decreased p16^INK4A^ levels in thymomas compared to NTs *in vivo* argued for a role of the increased cFLIP expression in attenuation of senescence in thymomas.

**Figure 4:**
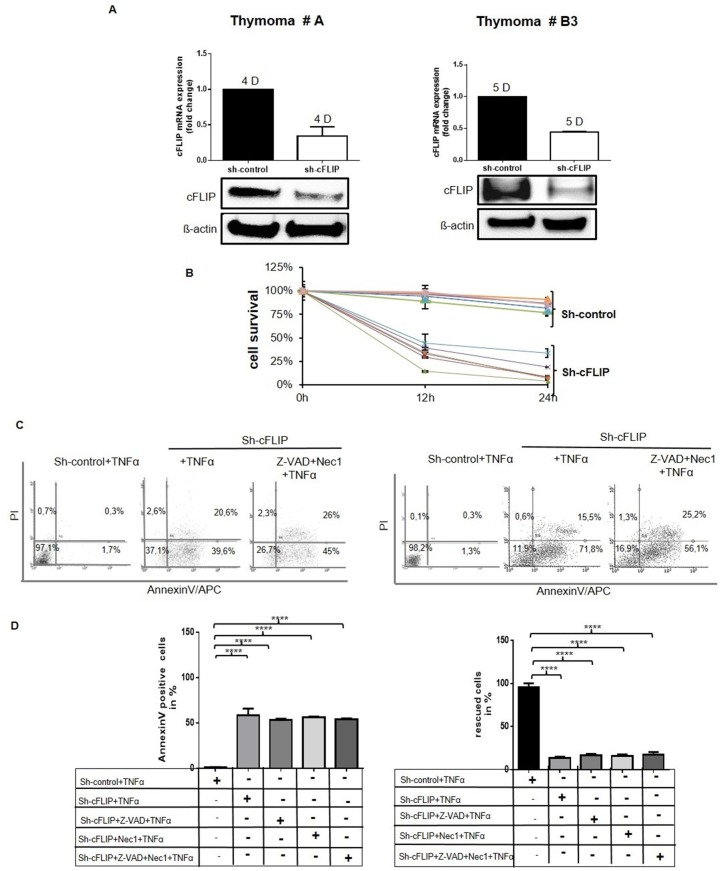
Sensitization of cultured TECs for TNF*α*-induced cell death following sh-cFLIP mediated knockdown pTECs from 2 different thymoma subtypes (A and B3) were transfected with either pU6^neo^-sh-cFLIP or scramble pU6^neo^-sh control plasmid **A.** Efficiency of cFLIP knockdown was measured 24 hours after transfection by real time PCR and western blot analysis. **B.** MTT assay reveals reduced pTECs viability 12 and 24 hours after transfection on treatment with 100ng/ml TNF*α* for 1 hour. **C.** Cell death was measured by AnnexinV-APC/PI flow cytometry: primary TECs were pretreated with either 50μM pan-caspase inhibitor Z-VAD-FMK or 100μM Nec1 or with both of these cell death inhibitors for one hour prior to transfection with cFLIP-shRNA (for 24 hours). Subsequently, pretreated cells and various control cells were treated with 100ng/ml TNF*α* for 1 hour. **D.** Left diagram: Representation of apoptotic cells (lower right region on the dot blot of figure C); right diagram: Rescue of cells (lower left region on the dot blot of figure C) because of caspase inhibition. The results shown are representative of three independent experiments.

To test this hypothesis, cFLIP RNA and protein levels were downregulated in 2 to 4 day-old pTECs by cFLIP shRNA (Figure 4A). Suppression of cFLIP for 12 and 24 hours followed by TNF*α* treatment decreased cell viability of pTECs to 50-80% and 75-95%, respectively compared to mock-transfected pTECs (Figure 4B). The thymic carcinoma cell line, 1889c, and HaCaT keratinocytes showed a similar reduction of cell survival upon cFLIP knockdown (Supplementary Figure 4A-4B). However, following cFLIP knockdown, only 1889c cells and HaCaT cells but not pTECs could be rescued from TNF*α*-induced cell death through the pan-caspase inhibitor Z-VAD-FMK or the necroptosis inhibitor, Necrostatin-1 (Nec1) (Figure 4C–4D und Supplementary Figure 4B-4D). Enforced cFLIP downregulation in pTECs was accompanied by p16^INK4A^ upregulation and accelerated senescence (Supplementary Figure 5A). Taken together, cFLIP counteracts senescence in neoplastic pTECs and prevents their death by a mechanism that is independent of caspases and the necroptosis inducer, RIPK1.

### NF-κB inhibition downregulates cFLIP expression in neoplastic thymic epithelia cells

**Figure 5:**
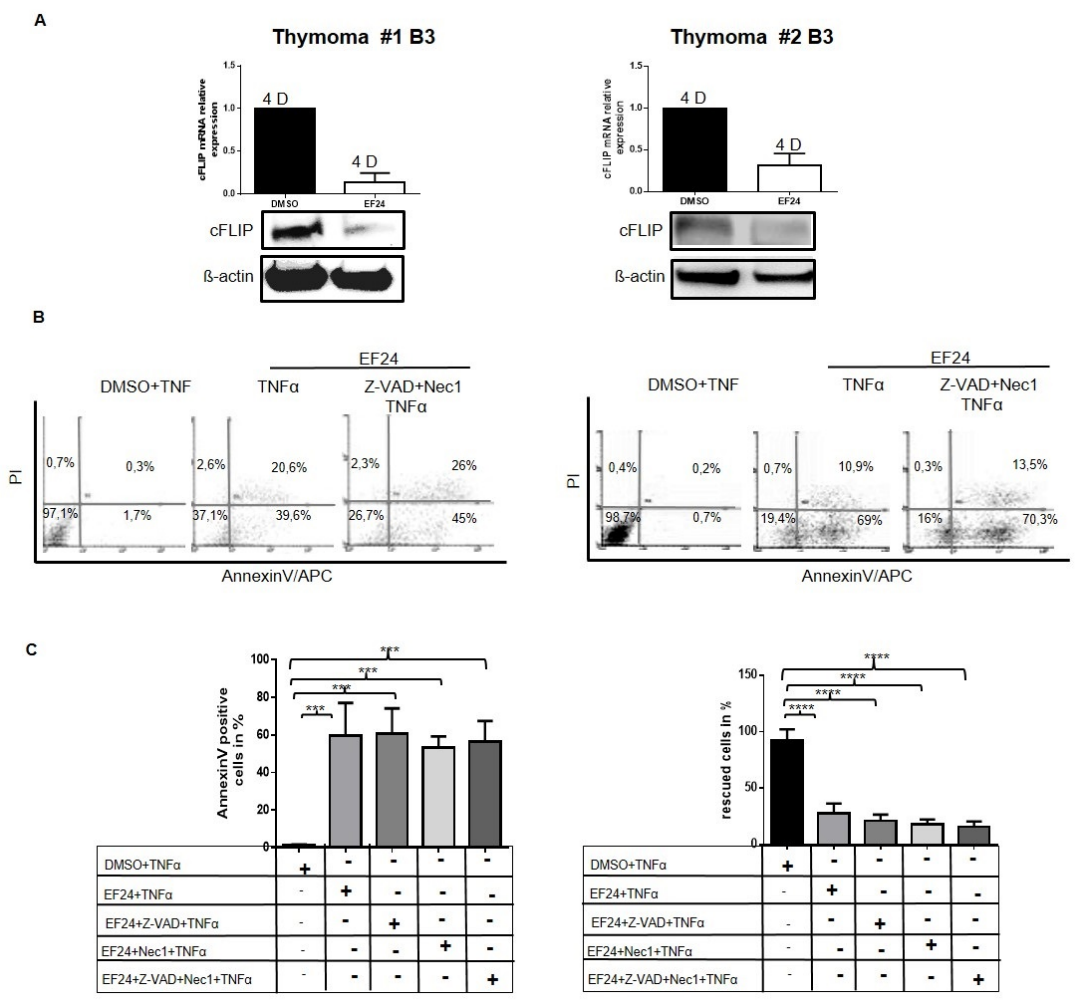
Cultured TECs are highly sensitive towards NF-κB inhibition and can not be rescued from cell death by inhibition of caspases and necroptosis pTECs from two B3 thymomas were first pretreated with Z-VAD-FMK for 1 hour before they were treated in addition with the NF-κB inhibitor, EF24 for 12hours Cells were then stimulated with 100ng/ml TNF*α for one hour*. RNA and proteins were analyzed for cFLIP expression using real time PCR and western blot **A.** (2 type B3 thymoma pTECs are shown). Cell death was determined with Annexin V/APC/PI using flow cytometry **B.** Dead cells represented 59.70%+/-10.% of pTECs compared to control cells (DMSO and TNF*α* treated) (^**^p=0.0043) (**C**, left diagram). Rescued cells after pan-caspase and necroptosis inhibitions in pTECs (**C**, right diagram: no significant effect of caspase inhibition). Results in C, diagrams represent the mean +/-SEM of duplicate experiments from freshly thawed cells. D, days in culture.

Since cFLIP expression is driven by activated NF-κB (p65) in some non-thymic tumors [11, 14], we treated neoplastic pTECs and control cell lines with the NF-κB inhibitor, EF24 [15] (Figure 5). This treatment reduced cFLIP mRNA and protein expression levels (Figure 5A) and facilitated TNF*α*-induced cell death in neoplastic pTECs of two B3 thymomas tested in passage 1 and 2 (Figure 5B and Figure 5C, left diagram, 2^nd^ column). Furthermore, EF24 treatment induced premature senescence in terms of X-Gal staining and accelerated p16 overexpression (data not shown). Death of neoplastic pTECs following NF-κB inhibition could not be prevented by either the pan-caspase inhibitor Z-VAD-FMK or Necrostatin 1 or both inhibitors combined (Figure 5C, right diagram, 2^nd^ column). By contrast, the EF24 treated control thymic carcinoma cell line, 1889c, and HaCaT cells could be partially rescued from TNF*α*-induced cell death by either Z-VAD-FMK or Necrostatin1 or both inhibitors (Supplementary Figure 5A). Therefore, like cFLIP knockdown, NF-κB inhibition can facilitate TNF*α*-induced cell death of pTECs by a caspase and RIPK1 independent mechanism.

### cFLIP knockdown and NF-κB inhibition induce autophagy in pTECs

**Figure 6:**
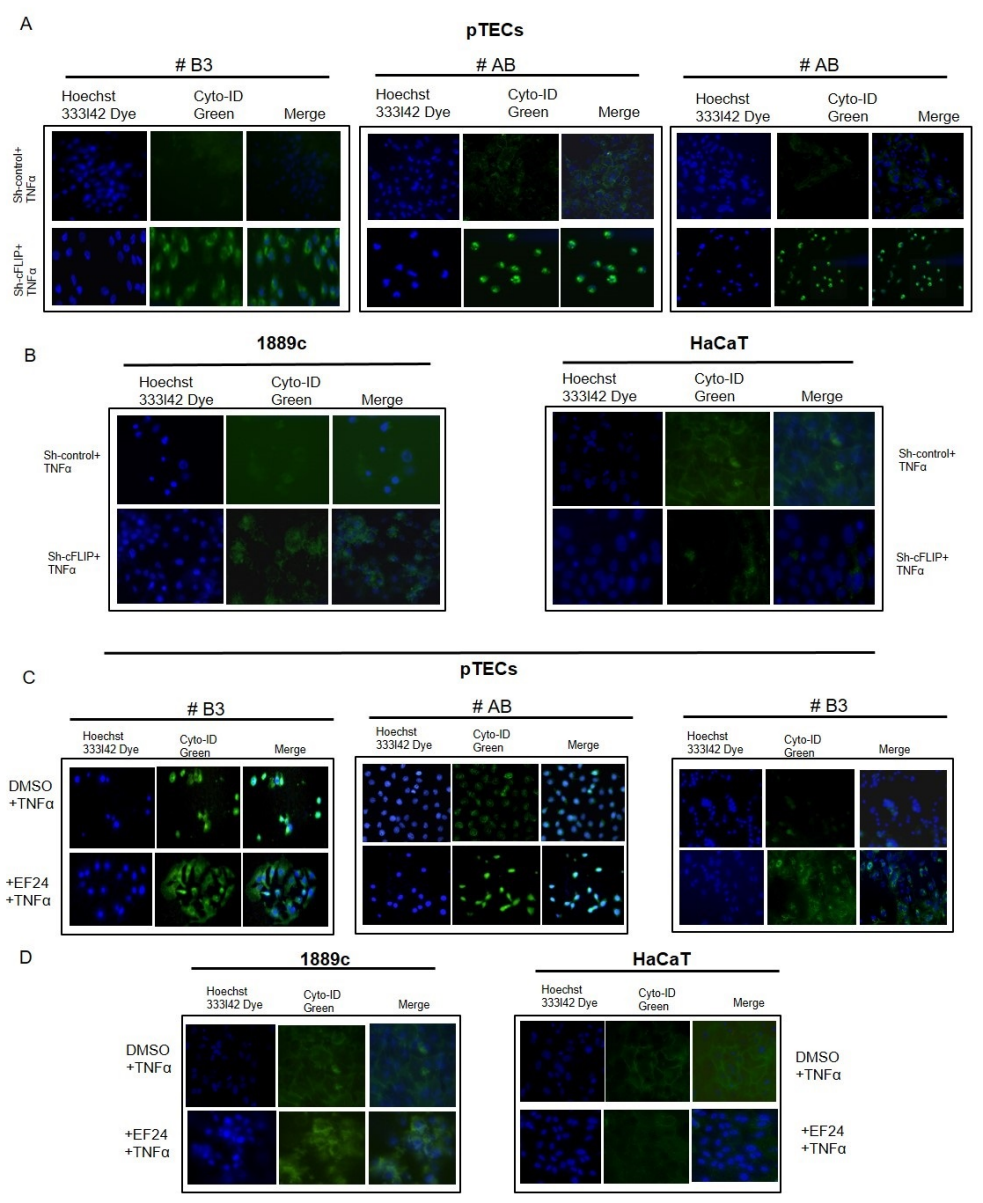
Sensitivity of sh-cFLIP transfected and EF24 treated pTECs to TNF*α*-induced autophagy Visualization of autophagic vacuoles in 4-day-old pTECs derived from one type AB and one B3 thymoma and in 1889c and HaCaT cells The cells were transfected using sh-cFLIP or sh-control RNA for 12h (pTECs) or 48h (1889c, HaCaT)) or treated with 3μM (1889c, HaCaT) and 1μM EF24 (pTECs) for 24h followed by 100ng/ml TNF*α* for 1 hour. After washing with PBS, cells were incubated with Cyto-ID^®^ Green dye for 30mn by 37°C. Green signals indicate Cyto-ID® Green labelled autophagic vesicles. Nuclei were counter-stained with Hoechst 33342 dye (blue).

Since autophagy can lead to caspase- and necroptosis-independent cell death [16], we next tested the impact of cFLIP knockdown on autophagy. Autophagic vacuoles were detected already 24 hours after shRNA-mediated cFLIP knockdown in all cultured pTECs of thymomas (n=3) (Figure 6A) but only after 48 hours in a minority of 1889c TC cells (Figure 6B). HaCaT cells showed no autophagic vacuoles (Figure 6B). Similar to cFLIP knockdown, the NF-κB inhibitor EF24 at concentrations that reduce cFLIP expression increased the number of autophagic vacuoles in pTECs and the control cell line 1889c but not in HaCaT cells (Figure 6C and 6D). This suggested that cFLIP interfered with autophagy in pTECs and, therefore, might be a candidate protein to attenuate autophagy-dependent cell death mechanisms.

### Blocking autophagy in cFLIP^low^ pTECs attenuates TNF*α*-induced cell death

**Figure 7:**
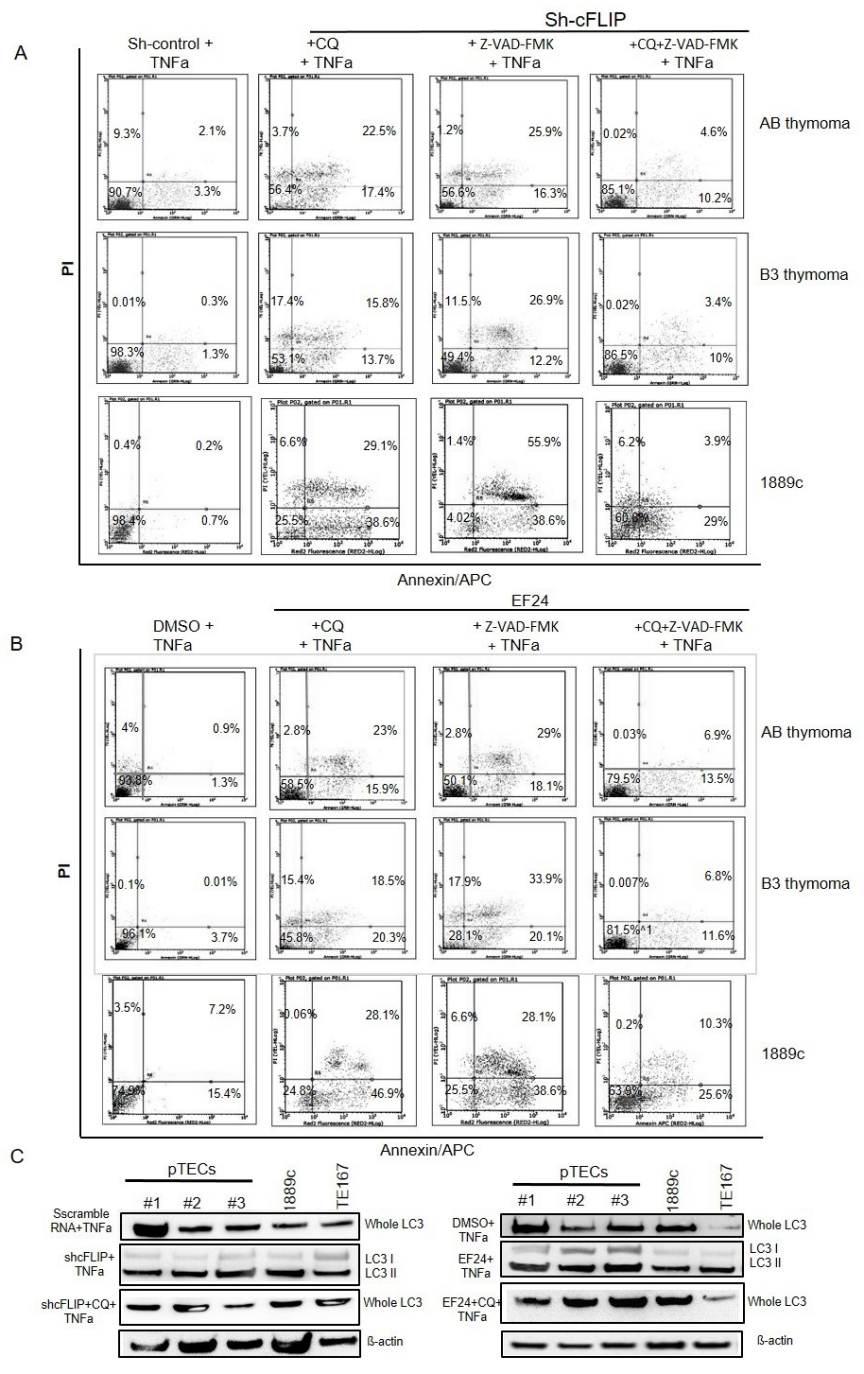
Attenuation of TNFα induced cell death by blocking autophagy and apoptosis in sh-cFLIP transfected and EF24 treated pTECs Autophagy inhibition by chloroquine (CQ) treatment pTECs from one AB thymoma, one B3 thymoma, thymic carcinoma 1889c and TE167 rhabdomyosarcoma cell lines were pretreated either with CQ 25M μM or 50μM Z-VAD-FMK or both agents for 1 hour before they were transfected with sh-cFLIP or treated with the NF-κB inhibitor, EF24 for 12hours. Cells were then stimulated with 100ng/ml TNF*α for one hour*: Cell death was determined with Annexin V-APC/PI using flow cytometry. **A.** Cell death in sh-cFLIP transfected pTECs of pTECs from AB and B3 thymomas) and TC 1889c cell line. **B.** Cell death in EF24 treated pTECs and TC 1889c cell line. **C.** Expression levels of LC3 and cleaved LC3 (LC3I and II) proteins in sh-cFLIP transfected (left figure) and EF24 treated cells (right figure). Note blockade of LC3 cleavage by CQ. ß-Actin was used as loading control.

To investigate whether autophagy blockade could block TNF*α* induced cell death, pTECs of two AB, one B2 and one B3 thymoma were pre-treated with either 25μM of the autophagy inhibitor, chloroquine (CQ), or 50μM of the pan-caspase inhibitor, Z-VAD-FMK or a combination of both agents before cFLIP knockdown or EF24 treatment. Pretreatment with either CQ or Z-VAD-FMK alone did not significantly block TNF*α* induced cell death of sh-cFLIP transfected pTECs and 1889c cell line (Figure 7A) and EF24 treated pTECs and 1889c cell line (Figure 7B), although autophagy was efficiently blocked by CQ as measured by western blot of LC3I/LC3II expression (Figure 7C) [17]. However, combined application of CQ and Z-VAD-FMKa rescued significant numbers of neoplastic pTECs and thymic carcinoma 1889c cells from TNF*α* induced cell death. These findings suggest that cFLIP fosters the survival of neoplastic pTECs by its dual inhibitory impact on apoptosis and autophagy.

## DISCUSSION

The main new findings here are i) higher expression of cFLIP in thymomas and TSCCs compared to NT *in vivo*; ii) delayed decline of cFLIP levels and delayed senescence in cultured neoplastic pTECs compared to normal pTECs; iii) regulation of cFLIP expression through NF-κB signaling in neoplastic pTECs; and iv) cell death induction through autophagy and apoptosis by cFLIP knockdown and NF-κB inhibition in pTECs.

### Relevance of epithelial cFLIP overexpression in TETs

Genes that regulate the ‘intrinsic’ apoptotic pathway, such as members of the BCL-2 and IAP (inhibitor of apoptosis) families, have been implicated in the development of TETs for long, and their overexpression heralds a poor prognosis [9, 18, 19]. Genetic gains of BCL-2 family genes belong to the commonest genetic abnormalities in TETs, and BCL-2 and BIRC3 overexpression are almost consistent features of TCs [9, 19]. By contrast, increased expression of cFLIP, i.e. a blocker of the ‘extrinsic’ death receptor pathway [11] has not been described in TETs so far. Since survival of primary cell cultures established from thymomas was significantly attenuated by TNF*α* treatment only after cFLIP knockdown, increased cFLIP expression appears to be functionally relevant. Together with increased expression of BCL2 and IAP family members in TETs [9, 19] the current findings imply that most TETs suffer from a block of both intrinsic and extrinsic cell death pathways, offering a potential explanation for the resistance of most unresectable TETs to current treatments [2].

TETs share high cFLIP expression levels mostly CFLIP_L_ with various solid and hematopoietic tumors [20, 21] but not with cutaneous squamous cell carcinomas (CSQCC) [22]. This is remarkable, since CSQCC and TETs are the only cancers that commonly express the thymus-‘specific’ transcriptional master regulator and survival factor, FOXN1 [23]. Since cFLIP expression levels are decreased in CSQCC compared to normal tissues, TRAIL therapy has been considered effective and safe for patients with cFLIP^low^ CSQSC [22]. By contrast, our results suggest that TRAIL treatment alone might not be effective in cFLIP^high^ TETs.

Increased expression of the large variant of cFLIP cFLIP_L_ and additionally increases expression of the small cFLIP variant cFLIP_S_ in B2 thymoma subtype (Figure 1) occurred across the spectrum of indolent to aggressive thymoma subtypes and highly malignant TSCC suggesting that cFLIP is an important regulator of TEC homoeostasis rather than driver of aggressiveness. This is also illustrated by our new finding that cFLIP appears to be involved in thymic involution: First, cFLIP expression showed an age-dependent decline in NT but not TETs *in vivo*. Second, *spontaneous* cFLIP decline in normal and neoplastic TECs showed different kinetics *in vitro*: while pTEC cultures of NTs showed rapidly declining cFLIP expression levels that were accompanied by early strong signs of senescence. Cultured neoplastic TECs maintained their increased cFLIP levels much longer (mostly cFLIP_L_ because of the isolated neoplastic TECs, which were from AB and B3 Thymoma, expressing only cFLIP_L_ (Figure 1)). However, when cFLIP levels dropped in neoplastic TECs after prolonged culture, this was again accompanied by epithelial senescence and growth arrest. Third, *forced* cFLIP downregulation by RNA interference or NF-κB inhibition led to reduced proliferation of neoplastic TECs, accelerated senescence and cell death. Since blockade of the extrinsic apoptotic pathway delays thymic involution in mice [24], conditional cFLIP overexpression and knockout in murine TECs *in vivo* may give hints to the role of cFLIP in thymoma development and thymic involution, respectively. cFLIP_S_ overexpression in B2 thymoma (Figure 1) suggests that the small cFLIP variant could play a role in the development of the B2 thymomen, how far cFLIP_S_ in involved in blocking the thymic senescence and involution muss be investigated further in TECs isolated from these neoplastic tissues of B2 thymoma. Fourth, declining cFLIP levels and subsequent senescence of cultured normal and neoplastic TECs were accompanied by upregulation of CDKN2A/p16^INKA^ that has been implicated in thymic epithelial stem cell biology and thymic aging [24, 25].

Absence of p16 expression is common in many tumors, occurs in 70% of TETs and is associated with poor prognosis [19]. Rarely, p16 deficiency in TETs results from homozygous loss of the CDKN2A locus at 9p21.3 [19]. This was not the case in any TET that we studied functionally, since CDKN2A transcription always increased during thymoma epithelial cell culture. Other known regulatory mechanisms of CDKN2A expression, such as methylation [25, 26], and miR-24 [26, 27] have not been studied in TETs. Therefore, it is unknown whether increased expression of cFLIP is involved in the observed downregulation of 16^INKA^ in TETs.

### Autophagy induction in thymoma TECs by cFLIP knockdown

In terms of cell death, cFLIP can prevent apoptosis, anoikis [28], necroptosis [29, 30]. c-FLIP_L_ but not cFLIP_S_ attenuates autophagy by directly acting on the autophagy machinery by inhibiting Atg3 binding to LC3, thereby decreasing LC3 processing [30, 31]. Unexpectedly, in all four primary thymoma epithelial cell cultures tested, cFLIP knockdown induced autophagosome formation and rendered pTECs sensitive to TNF*α*-induced cell death that could not be prevented by single agent pan-caspase and necroptosis inhibition. This suggests that cFLIP in thymomas prevents epithelial cell death mainly through blockade of both apoptosis and autophagy. By contrast, cFLIP-depleted cells of the 1889c thymic carcinoma cell line could partially be rescued from TNF*α*-induced cell death by inhibitors of caspases, necroptosis and autophagy, suggesting that cFLIP in 1889c carcinoma cells may regulate a broader spectrum of cell death-inducing targets. These studies must be extended to primary thymic carcinoma cells (that were not available to us) in order to learn whether cFLIP-regulated cell death pathways are really different between thymomas and TCs. Since TNF*α* treatment reduced survival of cFLIP-depleted, thymoma-derived pTECs by cFLIPshRNA or EF24 compared to untreated controls (Figure 5C and 7C), cell death control by intrinsic pathways is strongly operative in TETs and may need attention in future therapeutic trials for thymoma patients (see below).

### Impact of NF-κB downregulation on cFLIP expression and TEC survival

cFLIP expression is regulated by a broad spectrum of mechanisms, including histone acetylation [32] DNA damage and ubiquitination [33] miR-375 [34] sonic hedgehog and myc signalling [32, 35]; and Hsp90 [36, 37]. Here we focussed on NF-κB-driven cFLIP expression because it is common in many cancers [21, 38] Indeed, the NF-κB inhibitor, EF24 elicited cFLIP downregulation in primary thymoma TECs and 1889 thymic carcinoma cells [13] (Figure 4A and Supplementary Figure 6A) and sensitized them to TNFα induced cell death (Figure 4B and 4C and Supplementary Figure 6B and 6C). Like cFLIP knockdown, NF-κB blockade induced both apoptosis and autophagy in 1889c TC cells (Supplementary Figure 7 and Figure 7) and in primary thymoma-derived TECs (Figure 4; Figure 5 and Figure 7). Whether NF-κB expression in neoplastic TECs is driven by oncogenic mechanisms [38] or echoes the epithelial NF-κB signaling that is operative during early thymus development [39] is unknown.

### Translational perspective

Because TETs show variable and often combined increased expression of cFLIP, BCL2 and IAP family members [9, 19], TETs appear as promising candidates for trials testing novel inhibitors of multiple anti-apoptotic proteins [40]. Accordingly, anti-cell death signatures are candidate companion tissue-based biomarkers. Furthermore, therapeutic trials may also investigate blockers of NF-κB signaling [41] and new heat shock protein (HSP) inhibitors with improved drug tolerability [36], because cFLIP and autophagy-related proteins are clients of HSP90 [36] and HSP90 is expressed in TETs [37, 42]

Finally, expression of cFLIP by cancer cells has been described as an immune escape mechanism [43]. Therefore, cFLIP expression in TETs appears a reasonable adjunct biomarker in immune checkpoint inhibitor trials that are underway in light of the recent detection of high levels of PD-L1 in thymomas and TCs [44, 45] as IAP inhibitors, which were found to mimic co-stimulatory signalling in T cells [46] and the small-molecule IAP antagonists, such as LCL161, have entered clinical trials for their ability to induce tumor necrosis factor (TNF)-mediated apoptosis of cancer cells in multiple myeloma, This effect was not a result of direct induction of tumor cell death, but rather of upregulation of tumor-cell-autonomous type I interferon (IFN) signaling and a strong inflammatory response that resulted in the activation of macrophages and dendritic cells, leading to phagocytosis of tumor cells and notably, combination of LCL161 with the immune-checkpoint inhibitor anti-PD1 was curative in all of the treated mice [47].

In complementary work, a tumor cell line that was selected for resistance to immunotherapy upregulated AKT compared with sensitive wild-type cells, and this was associated with increased levels of BIRC2 and BIRC3, BCL-2 and BCL-XL [48].

## MATERIALS AND METHODS

### Patients

Characteristics of the patients, 82 (Supplementary Table 1) biopsies of thymomas and TSCCs and of 20 non-neoplastic thymuses are summarized in Table 1.

### Primary thymic epithelial cells (pTECs) and cell lines

pTECs were prepared and cultured as described [9]. Shortly, cell suspensions were prepared by several rounds of collagenase II digestion of tissue fragments, and grown at 37°C in a 5% CO_2_ on uncoated 10 cm tissue plastic dishes (Becton & Dickinson, Heidelberg, Germany) in RPMI 1640 with 4.5 g/L glucose, 25 mM Hepes, 200 mM L-Glutamine, 50 U/ml penicillin, 50 μg/ml streptomycin and 10% calf serum (PAA Inc., Freiburg, Germany). The medium was changed every 4 days. Epithelial cell content of primary cell cultures was determined by anti-EpCAM immunofluorescence (clone 4G10 Abcam, Heideberg, Germany) (Supplementary Figure 1A) and flow cytometry (Supplementary Figure 1B). Cell cultures with <50% EpCAM(+) cells were discarded. After 4-7 days, sub-confluent cells were trypsinized, and pTECs were split up for passaging, X-Gal staining, flow cytometry, isolation of DNA, RNA and proteins, and storage in liquid nitrogen. The thymic carcinoma cell line 1889c [13] was also grown in the above medium and has been kindly provided by Prof Ralf J. Riecker (Institute of Pathology, University Hospital, Heidelberg, Germany) in 2011. The immortalized keratinocyte cell line HaCaT was cultured in DMEM medium. (The cell lines (1889c and HaCat) were authentified in January 2016 by STR (short tandem repeat) Analysis in the cell line authentification service in Heidelberg, Germany).

### Cell proliferation assay

Mitochondrial dehydrogenase activity as surrogate proliferation marker was measured by MTT assay 12h after 3-(4, 5-Dimethyl-2-thiazolyl)-2, 5-diphenyl-2H-tetrazolium bromide treatment of cells at 50% confluency according to the manufacturer's protocol.

### Inhibition of nuclear factor-κB signalling by EF24

2×10^4^ cells/well were grown in 12-well plates and treated for 24 h with the NF-κB inhibitor, EF24 (3,5-Bis (2 flurobenzylidene) piperidin-4-one) dissolved in DMSO [15] at 1μM (pTECs), 6μM (1889c) and 3μM (HaCaT). Controls were cultured in DMSO only. cFLIP expression was measured by real time PCR and western blot, cell death by flow cytometry.

### Cell death analysis by flow cytometry

pTECs, 1889c and HaCaT cells were seeded at 2×10^4^ to 4×10^4^ per well in 48-well microtiter plates and pre-treated either with the caspase inhibitor, z-VAD-fmk (Promega, Germany) (50μM pTECs and HaCaT and 100μM 1889c) or with the RIPK1 inhibitor, necrostatin-1 (Nec-1, 50μM; DB Bioscience, Heidelberg, Germany) [49] or with both z-VAD-fmk and Nec-1 for 1 hour, then treated with EF24 for 24 hours or transfected with sh-cFLIP for 48 hours. Lastly, cell death was induced using 100 ng/ml human TNF*α* (RD Systems, Wiesbaden, Germany) and quantified using AnnexinV/APC/PI (DB Bioscience, Heidelberg, Germany) by fluorescence-activated cell sorting (guava easyCyte™) as described [50].

### Detection of autophagy

Autophagic vacuoles were identified using the Cyto-ID^®^ Autophagy Detection Kit (ENZO, Germany) in live pTECs and 1889c TC cells according to the manufacturer's protocol. Briefly, cells were transfected with shRNAcFLIP or scramble shRNA for 48 h or treated with EF24 for 24 hours and labelled with Cyto-ID dye for 30 min at 37°C. Cells were washed with 1X assay buffer, fixed with 4% paraformaldehyde in PBS for 10 min and visualized using a fluorescence microscopy. The unprocessed form of microtubule-associated protein 1 light chain 3 (LC3) which is involved in autophagosome formation during autophagy and the proteolytically cleaved forms (LC3I and LC3II) were detected by western blot analysis.

### Detection of senescence by X Gal staining

When cells were trypsinized for passaging, cells in parallel cultures were washed with PBS, fixed for 3-5 min in 4% paraformaldehyde in PBS at room temperature and stained with fresh senescence-associated-3-ß-galactosidase (SA-,3-Gal) staining solution (X-Gal staining) [51]. Treated cells were checked every hour after the beginning of X-Gal staining. Images were taken with a Leitz Laborlux 11 microscope at the time point when X-Gal staining became recognizable for the first time, which was monitored as the ‘time-to-senescence’ period.

### Western blot

Western blots were performed as described [9]. Membranes were probed with mouse anti-cFLIP (G11 D16A8, Cell Signaling, Heidelberg, Germany; against cFLIP_L_ and cFLIP_S_;), rabbit anti-LC3 (Acris, Herford, Germany) and rabbit anti-ß-actin (New England Biolabs Frankfurt, Germany) primary antibodies over night at 4°C. Bound antibodies were detected with secondary anti-mouse and anti-rabbit antibodies and visualized with a chemoluminescence detection kit (Pierce, Darmstadt, Germany).

### Real time PCR analysis

Reverse transcription was performed using 1 μg RNA First Strand Minus cDNA kit (Thermo Scientific, Heidelberg, Germany). The real time PCR was performed on the ABI STEP ONE PLUS TaqMan PCR System (Applied Biosystems) using FAST SYBR Green master mix (Applied Biosystems).

The relative quantification was calculated using the ΔΔCt method with GAPDH as internal control. The Primer sets used were forward 5’-CACTGAAAGTCCCCGTCAAC-3’ and reverse 5-’CGTGCTGTGTACCTGCCCAAT-3’ for cFLIP and forward 5’-TCGACAGTCAGCCGCATCT-3’ and reverse 5’-CCGTTGACTCCGACCTTCA-3’, for GAPDH: 5’-TCGACAGTCAGCCGCATCT-3’ forward and 5’-CCGTTGACTCCGACCTTCA-3’, for cytokeratin 19 (CK19): 5’- CATGACTTCCTACAGCTATGC forward and 5’ CGCGAAGAGGACTGGACGGTT -3’ reverse and for p16: 5’-CCACCCTGGCTCTGACCAT-3’ forwards and 5’-GCCACTCGGGCGCTG-3’ reverse.

### Statistical analysis

All statistical analyses were performed with GraphPad Prism V6.0 (GraphPad Software Inc, La Jolla, USA). Two-tailed student's *t*-test was used with p<0.05 and a confidence level of 95% (p<0.05 was considered significant) when comparing cFLIP gene expression levels in different groups of thymomas. A subsequent F-test was used to compare variances with p<0.05 at a confidence level of 95% (p<0.05) was considered as significant.

## SUPPLEMENTARY MATERIALS FIGURES AND TABLES



## References

[1] Ströbel P, Hohenberger P, Marx A (2010). Thymoma and thymic carcinoma: molecular pathology and targeted therapy. J Thorac Oncol.

[2] Travis WD, Brambilla E, Burke AP, Marx A, Nicholson AG (2015). Introduction to The 2015 World Health Organization Classification of Tumors of the Lung, Pleura, Thymus, and Heart. Introduction to The 2015 World Health Organization Classification of Tumors of the Lung, Pleura, Thymus, and Heart. J Thorac Oncol.

[3] Marx A, Pfister F, Schalke B, Saruhan-Direskeneli G, Melms A, Ströbel P (2013). The different roles of the thymus in the pathogenesis of the various myasthenia gravis subtypes. Autoimmun Rev.

[4] Meager A, Peterson P, Willcox N (2008). Hypothetical review: thymic aberrations and type-I interferons; attempts to deduce autoimmunizing mechanisms from unexpected clues in monogenic and paraneoplastic syndromes. Clin Exp Immunol.

[5] Ahmad U, Yao X, Detterbeck F, Huang J, Antonicelli A, Filosso PL, Ruffini E, Travis W, Jones DR, Zhan Y, Lucchi M, Rimner A (2015). Thymic carcinoma outcomes and prognosis: results of an international analysis. J Thorac Cardiovasc Surg.

[6] Petrini I, Meltzer PS, Kim IK, Lucchi M, Park KS, Fontanini G, Gao J, Zucali PA, Calabrese F, Favaretto A, Rea F, Rodriguez-Canales J, Walker RL (2014). A specific missense mutation in GTF2I occurs at high frequency in thymic epithelial tumors. Nat Genet.

[7] Gray DH, Seach N, Ueno T, Milton MK, Liston A, Lew AM, Goodnow CC, Boyd RL (2006). Developmental kinetics, turnover, and stimulatory capacity of thymic epithelial cells. Blood.

[8] Zettl A, Ströbel P, Wagner K, Katzenberger T, Ott G, Rosenwald A, Peters K, Krein A, Semik M, Müller-Hermelink HK, Marx A (2000). Recurrent genetic aberrations in thymoma and thymic carcinoma. Am J Pathol.

[9] Huang B, Belharazem D, Li L, Kneitz S, Schnabel PA, Rieker RJ, Körner D, Nix W, Schalke B, Müller-Hermelink HK, Ott G, Rosenwald A, Ströbel P, Marx A (2013). Anti-Apoptotic Signature in Thymic Squamous Cell Carcinomas - Functional Relevance of Anti-Apoptotic BIRC3 Expression in the Thymic Carcinoma Cell Line 1889c. Front Oncol.

[10] Safa AR, Day TW, Wu CH (2008). Cellular FLICE-like inhibitory protein (C-FLIP): a novel target for cancer therapy. Curr Cancer Drug Targets.

[11] Safa AR (2012). c-FLIP, a master anti-apoptotic regulator. Exp Oncol.

[12] Girard N, Ruffini E, Marx A, Faivre-Finn C, Peters S, ESMO Guidelines Committee (2015). Thymic epithelial tumours: ESMO Clinical Practice Guidelines for diagnosis, treatment and follow-up. Ann Oncol.

[13] Ehemann V, Kern MA, Breinig M, Schnabel PA, Gunawan B, Schulten HJ, Schlaeger C, Radlwimmer B, Steger CM, Dienemann H, Lichter P, Schirmacher P, Rieker RJ (2008). Establishment, characterization and drug sensitivity testing in primary cultures of human thymoma and thymic carcinoma. Int J Cancer.

[14] Shirley S, Micheau O (2013). Targeting c-FLIP in cancer. Cancer Lett.

[15] Kasinski AL, Du Y, Thomas SL, Zhao J, Sun SY, Khuri FR, Wang CY, Shoji M, Sun A, Snyder JP, Liotta D, Fu H (2008). Inhibition of IkappaB kinase-nuclear factor-kappaB signaling pathway by 3,5-bis(2-flurobenzylidene)piperidin-4-one (EF24), a novel monoketone analog of curcumin. Mol Pharmacol.

[16] Levine B, Kroemer G (2008). Autophagy in the pathogenesis of disease. Cell.

[17] Glick D, Barth S, Macleod KF (2010). Autophagy: cellular and molecular mechanisms. J Pathol.

[18] Hiroshima K, Iyoda A, Toyozaki T, Supriatna Y, Shibuya K, Shimamura F, Haga Y, Yoshida S, Fujisawa T, Ohwada H (2002). Proliferative activity and apoptosis in thymic epithelial neoplasms. Mod Pathol.

[19] Petrini I, Meltzer PS, Zucali PA, Luo J, Lee C, Santoro A, Lee HS, Killian KJ, Wang Y, Tsokos M, Roncalli M, Steinberg SM, Wang Y, Giaccone G (2012). Copy number aberrations of BCL2 and CDKN2A/B identified by array-CGH in thymic epithelial tumors. Cell Death Dis.

[20] Ryu BK, Lee MG, Chi SG, Kim YW, Park JH (2001). Increased expression of cFLIP(L) in colonic adenocarcinoma. J Pathol.

[21] Mathas S, Lietz A, Anagnostopoulos I, Hummel F, Wiesner B, Janz M, Jundt F, Hirsch B, Jöhrens-Leder K, Vornlocher HP, Bommert K, Stein H, Dörken B (2004). c-FLIP mediates resistance of Hodgkin/Reed-Sternberg cells to death receptor-induced apoptosis. J Exp Med.

[22] Leverkus M, Neumann M, Mengling T, Rauch CT, Bröcker EB, Krammer PH, Walczak H (2000). Regulation of tumor necrosis factor-related apoptosis-inducing ligand sensitivity in primary and transformed human keratinocytes. Cancer Res.

[23] Nonaka D, Henley JD, Chiriboga L, Yee H (2007). Diagnostic utility of thymic epithelial markers CD205 (DEC205) and Foxn1 in thymic epithelial neoplasms. Am J Surg Pathol.

[24] Dumont-Lagacé M, Brochu S, St-Pierre C, Perreault C (2014). Adult thymic epithelium contains nonsenescent label-retaining cells. J Immunol.

[25] Hirabayashi H, Fujii Y, Sakaguchi M, Tanaka H, Yoon HE, Komoto Y, Inoue M, Miyoshi S, Matsuda H (1997). p16INK4, pRB, p53 and cyclin D1 expression and hypermethylation of CDKN2 gene in thymoma and thymic carcinoma. Int J Cancer.

[26] Lal A, Kim HH, Abdelmohsen K, Kuwano Y, Pullmann R, Srikantan S, Subrahmanyam R, Martindale JL, Yang X, Ahmed F, Navarro F, Dykxhoorn D, Lieberman J, Gorospe M (2008). p16(INK4a) translation suppressed by miR-24. PLoS One.

[27] Yajima N, Sakamaki K, Yonehara S (2004). Age-related thymic involution is mediated by Fas on thymic epithelial cells. Int Immunol.

[28] Tan K, Goldstein D, Crowe P, Yang JL (2013). Uncovering a key to the process of metastasis in human cancers: a review of critical regulators of anoikis. J Cancer Res Clin Oncol.

[29] Feoktistova M, Leverkus M (2015). Programmed necrosis and necroptosis signalling. FEBS J.

[30] Safa AR (2013). Roles of c-FLIP in Apoptosis, Necroptosis, and Autophagy. J Carcinog Mutagen.

[31] Lee JS, Li Q, Lee JY, Lee SH, Jeong JH, Lee HR, Chang H, Zhou FC, Gao SJ, Liang C, Jung JU (2009). FLIP-mediated autophagy regulation in cell death control. Nat Cell Biol.

[32] Bangert A, Cristofanon S, Eckhardt I, Abhari BA, Kolodziej S, Häcker S, Vellanki SH, Lausen J, Debatin KM, Fulda S (2012). Histone deacetylase inhibitors sensitize glioblastoma cells to TRAIL-induced apoptosis by c-myc-mediated downregulation of cFLIP. Oncogene.

[33] Stagni V, Mingardi M, Santini S, Giaccari D, Barilà D (2010). ATM kinase activity modulates cFLIP protein levels: potential interplay between DNA damage signalling and TRAIL-induced apoptosis. Carcinogenesis.

[34] Wang J, Huang H, Wang C, Liu X, Hu F, Liu M (2013). MicroRNA-375 sensitizes tumour necrosis factor-alpha (TNF-α)-induced apoptosis in head and neck squamous cell carcinoma in vitro. Int J Oral Maxillofac Surg.

[35] Kump E, Ji J, Wernli M, Häusermann P, Erb P (2008). Gli2 upregulates cFlip and renders basal cell carcinoma cells resistant to death ligand-mediated apoptosis. Oncogene.

[36] Henrich CJ, Brooks AD, Erickson KL, Thomas CL, Bokesch HR, Tewary P, Thompson CR, Pompei RJ, Gustafson KR, McMahon JB, Sayers TJ (2015). Withanolide E sensitizes renal carcinoma cells to TRAIL-induced apoptosis by increasing cFLIP degradation. Cell Death Dis.

[37] Breinig M, Mayer P, Harjung A, Goeppert B, Malz M, Penzel R, Neumann O, Hartmann A, Dienemann H, Giaccone G, Schirmacher P, Kern MA, Chiosis G, Rieker RJ (2011). Heat shock protein 90-sheltered overexpression of insulin-like growth factor 1 receptor contributes to malignancy of thymic epithelial tumors. Clin Cancer Res.

[38] Staudt LM (2010). Oncogenic activation of NF-kappaB. Cold Spring Harb Perspect Biol.

[39] Onder L, Nindl V, Scandella E, Chai Q, Cheng HW, Caviezel-Firner S, Novkovic M, Bomze D, Maier R, Mair F, Ledermann B, Becher B, Waisman A, Ludewig B (2015). Alternative NF-κB signaling regulates mTEC differentiation from podoplanin-expressing precursors in the cortico-medullary junction. Eur J Immunol.

[40] Braig S, Bischoff F, Abhari BA, Meijer L, Fulda S, Skaltsounis L, Vollmar AM (2014). The pleiotropic profile of the indirubin derivative 6BIO overcomes TRAIL resistance in cancer. Biochem Pharmacol.

[41] Park YH, Seo SY, Lee E, Ku JH, Kim HH, Kwak C (2013). Simvastatin induces apoptosis in castrate resistant prostate cancer cells by deregulating nuclear factor-κB pathway. J Urol.

[42] Gallerne C, Prola A, Lemaire C (2013). Hsp90 inhibition by PU-H71 induces apoptosis through endoplasmic reticulum stress and mitochondrial pathway in cancer cells and overcomes the resistance conferred by Bcl-2. Biochim Biophys Acta.

[43] Todaro M, Lombardo Y, Francipane MG, Alea MP, Cammareri P, Iovino F, Di Stefano AB, Di Bernardo C, Agrusa A, Condorelli G, Walczak H, Stassi G (2008). Apoptosis resistance in epithelial tumors is mediated by tumor-cell-derived interleukin-4. Cell Death Differ.

[44] Katsuya Y, Fujita Y, Horinouchi H, Ohe Y, Watanabe S, Tsuta K (2015). Immunohistochemical status of PD-L1 in thymoma and thymic carcinoma. Lung Cancer.

[45] Padda SK, Riess JW, Schwartz EJ, Tian L, Kohrt HE, Neal JW, West RB, Wakelee HA (2015). Diffuse high intensity PD-L1 staining in thymic epithelial tumors. J Thorac Oncol.

[46] Dougan M, Dougan S, Slisz J, Firestone B, Vanneman M, Draganov D, Goyal G, Li W, Neuberg D, Blumberg R, Hacohen N, Porter D, Zawel L, Dranoff G (2010). IAP inhibitors enhance co-stimulation to promote tumor immunity. J Exp Med.

[47] Chesi M, Mirza NN, Garbitt VM, Sharik ME, Dueck AC, Asmann YW, Akhmetzyanova I, Kosiorek HE, Calcinotto A, Riggs DL, Keane N, Ahmann GJ, Morrison KM (2016). IAP antagonists induce anti-tumor immunity in multiple myeloma. Nat Med.

[48] Vanneman M, Dranoff G (2012). Combining immunotherapy and targeted therapies in cancer treatment. Nat Rev Cancer.

[49] Geserick P, Hupe M, Moulin M, Wong WW, Feoktistova M, Kellert B, Gollnick H, Silke J, Leverkus M (2009). Cellular IAPs inhibit a cryptic CD95-induced cell death by limiting RIP1 kinase recruitment. J Cell Biol.

[50] Mosmann T (1983). Rapid colorimetric assay for cellular growth and survival: application to proliferation and cytotoxicity assays. J Immunol Methods.

[51] Dimri GP, Lee X, Basile G, Acosta M, Scott G, Roskelley C, Medrano EE, Linskens M, Rubelj I, Pereira-Smith O, Peacocke M, Campisi J (1995). A biomarker that identifies senescent human cells in culture and in aging skin in vivo. Proc Natl Acad Sci USA.

