# Supplementary material for: Increased cFLIP expression in thymic epithelial tumors blocks autophagy via NF-κB signalling
Source: Oncotarget. 2017 Feb 6;8(52):89580–94. doi: 10.18632/oncotarget.15929 (PMC5685693; doi:10.18632/oncotarget.15929)
Supplement: Supplementary file 1 [file oncotarget-08-89580-s001.pdf]

Increased cFLIP expression in thymic epithelial tumors blocks autophagy via NF-κB signalling

SUPPLEMENTARY MATERIALS

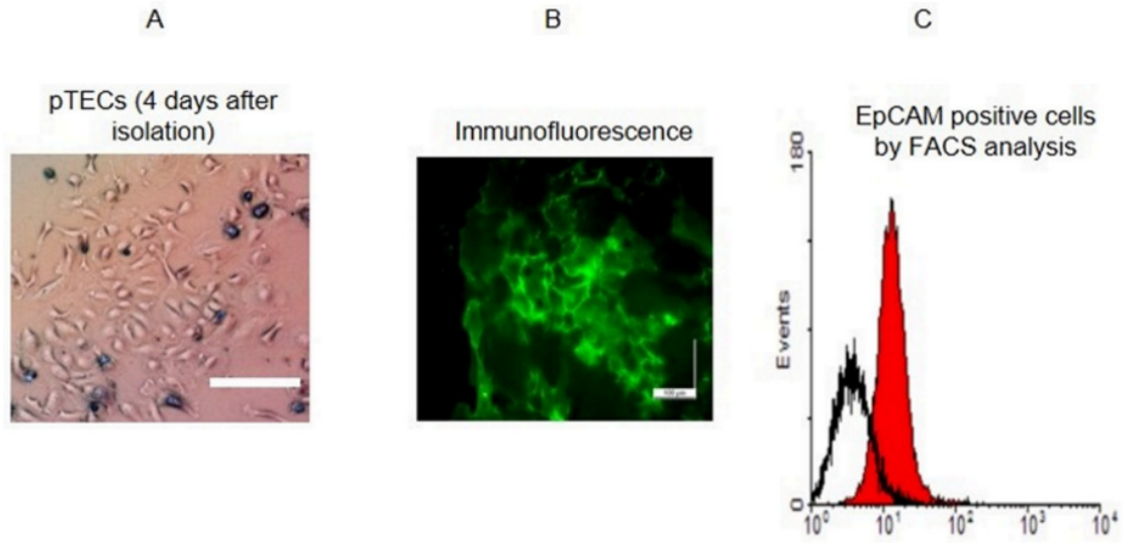

Supplementary Figure 1: *In vitro* characterization of freshly isolated primary thymic epithelial cells (pTECs). A. Phase-contrast microscopy of pTECs four days after tissue digestion. **B.** EpCAM identification in methanol-fixed pTECs by immunofluorescence (FITCS-conjugated anti-EpCAM antibody 4G10. **C.** Quantification of EpCAM-positive pTECs in primary TEC cultures by flow cytometry (antibody 4G10 (red); isotype control (black line)). Magnification x50 in (A) and x200 in (B).

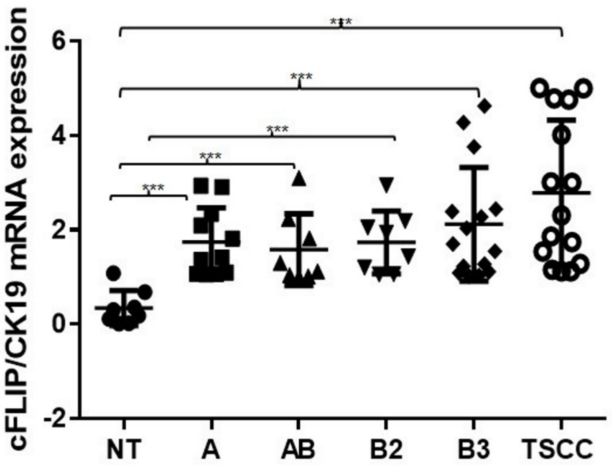

Supplementary Figure 2: Comparison of cFLIP and cytokeratin 19 mRNA expressions. Using real time PCR, mRNA levels of cFLIP were compared with levels of cytokeratin 19 (CK19) which is an almost consistently expressed thymic epithelial cell marker (that was detected in all cases studied here). Delta Ct of each sample in each scatter plot was calculated using CK19 as reference gene. The results represent the mean +/- SEM of triplicate measurements.).

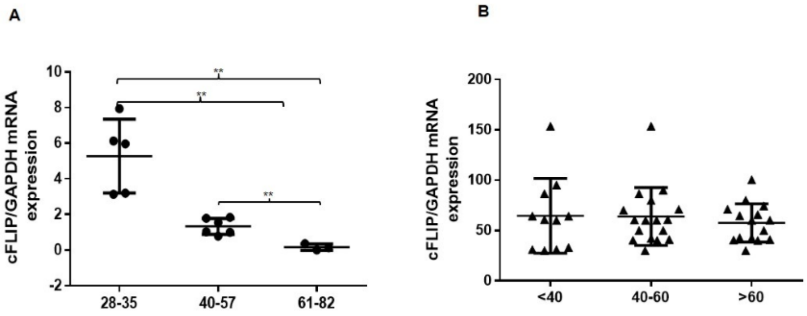

Supplementary Figure 3: cFLIP expression declines with age in normal thymuses (NTs, n=14) but not in thymic epithelial tumors (thymomas and TCs, n=43). **A.** Differences of cFLIP expression levels in NTs were significantly different between each of the three age groups (28-35, 40-57 and 61-82 years) (** = p <0.01). **B.** No age-dependent decline of cFLIP expression levels in thymomas and thymic carcinomas. Real time PCR measurements of whole tissue RNA extracts were performed in duplicates; results represent the mean +/- SEM.

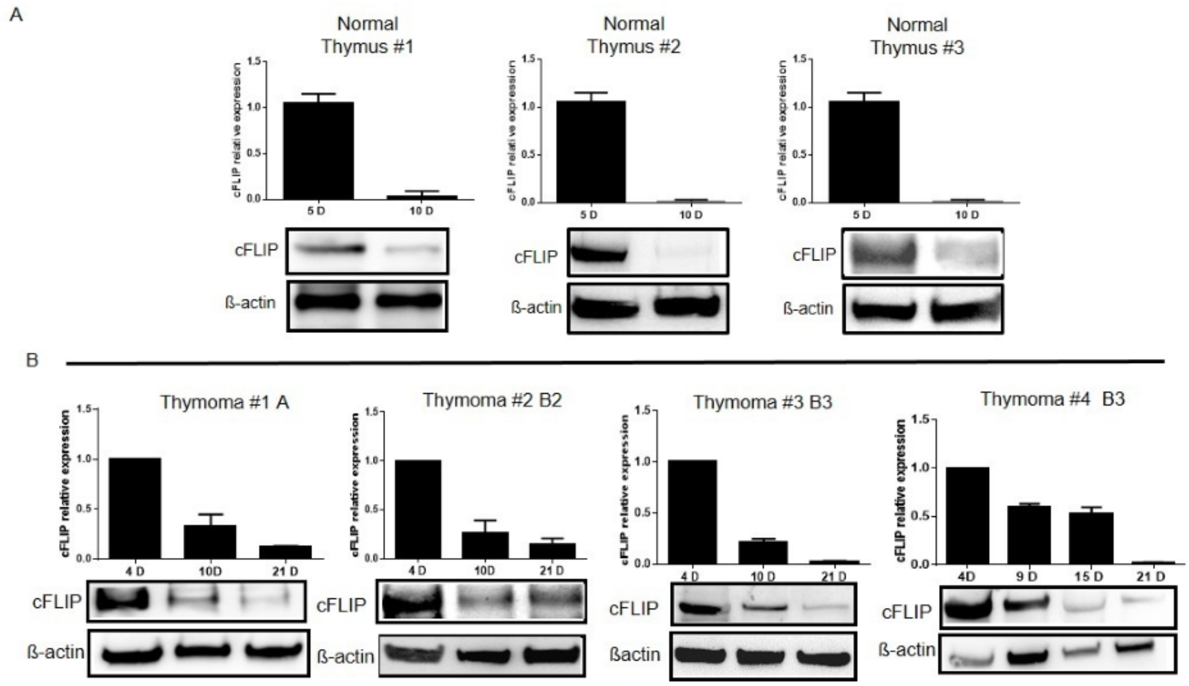

Supplementary Figure 4: Slower decline of cFLIP mRNA and protein levels in pTECs of more cases of thymomas and NTs (compare Figure 2) Expression levels of cFLIP on passaging of 3 primary epithelial cells from normal thymuses. **A.** compared to pTECs from 4 thymomas **B.** (All different from the pTECs shown in Figure 2). Real time PCR of triplicates measurements of two independent experiments with freshly thawed cells. The results represent the mean +/- SEM.

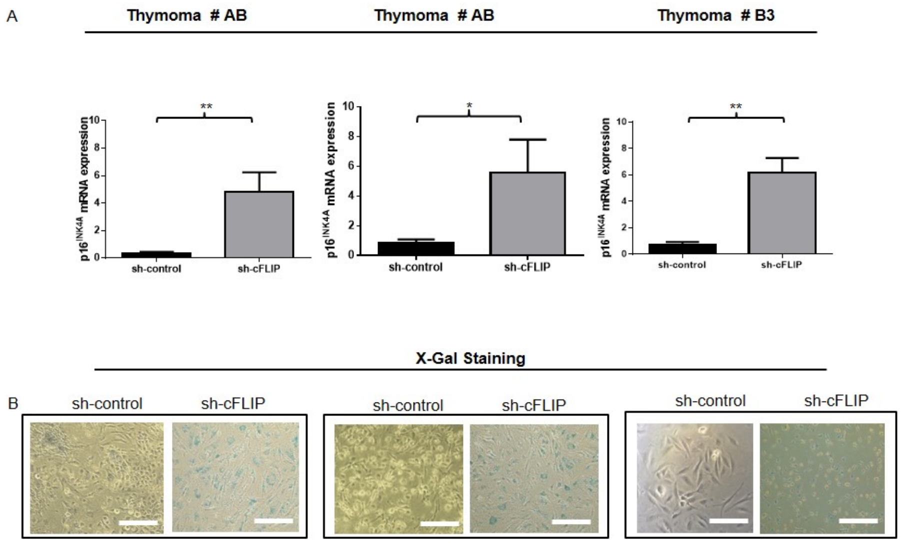

Supplementary Figure 5: Senescence detection and p16^{INK4A} expression in pTECs following sh-cFLIP mediated cFLIP knockdown. A. Increased p16^{INK4A} mRNA expression in sh-cFLIP transfected pTECs from 3 thymomas (2 AB and 1 B3) was detected by real time PCR. **B.** X-Gal staining from the same (Parallel cultures) cells for senescence detection (magnification x50). The results in (A) represent the mean +/- SEM.

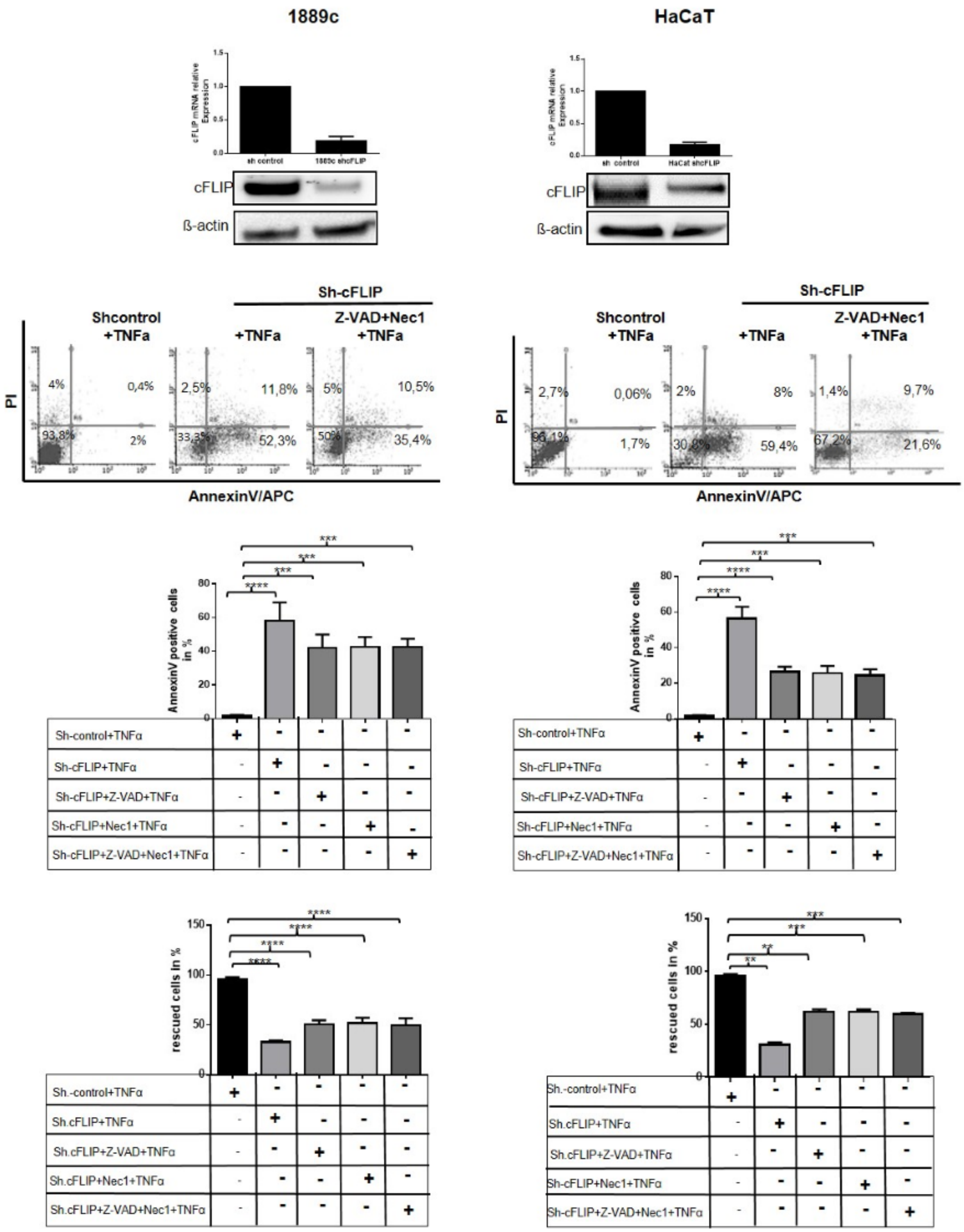

Supplementary Figure 6: Sensitivity of 1889c and HaCaT cell lines to cFLIP down regulation and their rescue from cell death by inhibition of caspases and necroptosis Thymic carcinoma 1889c (left part) and HaCaT (right part) cell lines were first pretreated Z-VAD-FMK, Nec1 or both agents one hour before transfection with pU6^{neo}-sh-cFLIP. Fourty eight hours after transfection the cell were stimulated using 100ng/ml TNFα. **A.** RNA and proteins were analyzed for cFLIP expression using real time PCR and western blot. **B.** Cell death was determined with annexinV-APC/PI using flow cytometry. **C.** Representative scheme of cell death (annexin V positive cells) The columns represent the dead (annexinVpositive) cells. **D.** Rescue of cells by caspase inhibition. The columns represent the cell survival (annexinV/APC PI negative cells, which cells were localized in lower left (LL) area of the dot blots in Supplementary Figure S4B. The results in (A) represent the mean +/- SEM of three independent experiments. Cells that were transfected with scramble sh-pU6^{neo} plasmid served as control.

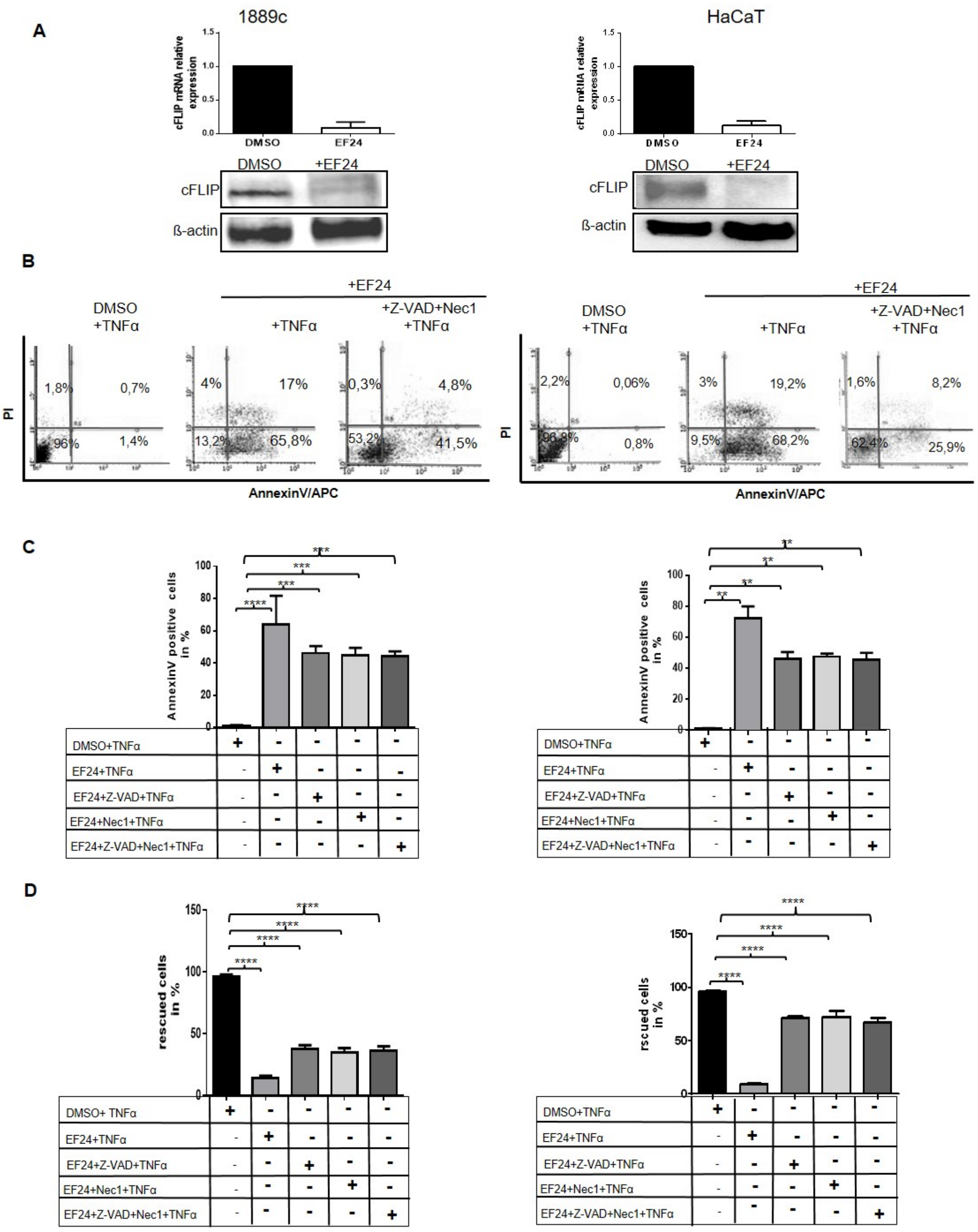

Supplementary Figure 7: Sensitivity of 1889c and HaCaT cell lines to NF-κB inhibition and their rescue from cell death by inhibition of caspases and necroptosis. Thymic carcinoma 1889c (left part) and HaCaT (right part) cell lines were first pretreated with the pan-caspase inhibitor, Z-VAD-FMK for 1 hour, followed by treatment with the NF-κB inhibitor, EF24 for 48 h (1889) or 24 h (HaCaT). Cells were then stimulated with 100ng/ml TNFα for one hour. RNA and proteins were analyzed for cFLIP expression using real time PRC and western blot **A.** Cell death was determined with AnnexinV-APC/PI using flow cytometry **B.** Dead cells presented 63.9%+/-10.2 ** PCR p=0.0036 in 1889c and 72.3+/-4.4%, **** p <0.0001 in HaCaT. **D.** Rescue from cell death through pan-caspase inhibition of 1889c (38.2%+/-1.15% *** p=0.0002) and HaCaT cells (55.6% +/-3.5% *** p=0.0001) (cells were localized in lower left (LL) area of the dot blots in (Supplementary Figure S5B). Three independent experiments were performed.

Supplementary Table 1: cFLIP/CK19 mRNA expression ratios of thymomas and TSCC

Thymoma & Thymic carcinoma	N=(56)	cFLIP/CK19 ratio	p-value
Type A	11	1.72 +/- 0.22	p=0.0001
Type AB	8	1.58 +/- 0.26	p=0.0001
Type B2	8	1.73 +/- 0.23	p=0.0001
Type B3	15	1.6+/- 0.25	p=0.0006
TSCC	15	2 +/- 0.30	p= 0.0003

P-values concern the differences to the ratios calculated for NTs (n=12; 0.33 +/-0.13).
